# Glomerulonephritis Associated With Infected Cardiac Pacemaker Lead Mimics Infective Endocarditis-Associated Glomerulonephritis With Resolution After Lead Removal: A Case Report and Literature Review

**DOI:** 10.7759/cureus.46471

**Published:** 2023-10-04

**Authors:** Jaime Said, Bridget Budny, Alexandra Sappington, Tiffany Caza, Ahmad O Rifai, Sarah Rifai, Kristin M Denig

**Affiliations:** 1 Medicine, Alabama College of Osteopathic Medicine, Dothan, USA; 2 Nephropathology, Arkana Laboratories, Little Rock, USA; 3 Nephrology, The Virtual Nephrologist, Inc., Lynn Haven, USA; 4 Nephrology, The Virtual Nephrologist, Inc., Panama City, USA

**Keywords:** infection, negative blood culture, infected lead, pacemaker lead infection-associated glomerulonephritis, pacemaker leads, infective endocarditis, glomerulonephritis

## Abstract

The incidence of cardiac pacemaker lead infections is increasing due to the rise in cardiac implantable device use. These infections mimic infective endocarditis (IE) and cause a variety of complications. However, there is a scarcity of knowledge regarding glomerulonephritis (GN) resulting from cardiac pacemaker-lead infections. This report describes a 71-year-old female who presented with GN associated with a cardiac pacemaker-lead infection. The patient was successfully treated with intravenous (IV) antibiotics, IV steroids, and early surgical removal of the cardiac pacemaker lead, resulting in the resolution of GN. Current guidelines do not address cardiac pacemaker lead infection-associated GN as an indication for lead removal. Given the success of our treatment approach and the rising incidence of cardiac pacemaker infections, we suggest the consideration of early surgical removal of the cardiac lead, in conjunction with antibiotics and steroids, for the treatment of cardiac lead infection associated with GN. Further research is necessary to determine the prevalence and optimal management of this complication.

## Introduction

Infective endocarditis (IE) is a severe clinical condition with a 20% mortality rate, despite treatment with antibiotics and surgical procedures [1,2]. Gram-positive cocci are the leading cause of IE, with Staphylococcus aureus primarily targeting native valves and coagulase-negative staphylococci infecting prosthetic valves and cardiac devices [1,3]. Common organisms causing subacute IE are Viridans streptococci, Enterococcus, S. epidermidis, and S. bovis [1,2]. Gram-negative bacteria and fungi are the causative agents in a small portion of cases [3].

IE can present as an acute or subacute disease [3]. Acute IE advances rapidly, typically due to a higher virulence pathogen [3]. A new-onset heart murmur in the setting of an acute illness with positive blood cultures is nearly pathognomonic for IE [3]. However, patients with subacute IE develop non-specific symptoms, and less than 50% of cases present with a new murmur [3]. A diagnosis of IE is made utilizing the Modified Duke Criteria as a clinical guideline, requiring either a histologically proven vegetation or a combination of various clinical and laboratory findings, including positive blood cultures, echocardiographic evidence, new onset regurgitation, predisposing history (heart condition or intravenous (IV) drug use), fever, and/or vascular or immunologic phenomena [2-6].

Cardiac pacemaker-lead infections can mimic IE clinically, often meeting modified Duke’s criteria and involving the same pathogens with an insidious onset [4]. The prevalence of this complication is increasing [7,8]. Between 1993 and 2006, the implantation of cardiovascular implantable electronic devices (CIED) increased by 96% [7,8], with the associated infection rate increasing over twofold [7,8]. Inflammatory reactions may be blunted in older populations, and a lower threshold for echocardiography is indicated for the detection of cardiac device infections [4].

Approximately one-third of bacterial endocarditis cases are complicated by acute kidney injury (AKI) resulting in increased morbidity and mortality [4,9,10]. Infective endocarditis-related glomerulonephritis (GN) makes up 6%-20% of all infection-related GN cases and primarily leads to the membranoproliferative type [11]. The frequency of AKI resulting from cardiac lead infections remains unknown.

It is well documented that indications for surgical intervention in IE include persistent bacteremia or fungemia despite antimicrobial therapy, recurrent embolic events, cardiac conduction defects, and sudden valvular dysfunction [2-6,12]. In the case of cardiac lead infections, the clinical practice guidelines recommend complete removal of the device in definitive cardiac device-related IE but do not elaborate on the connection between lead infection and GN [4,6,12]. We report a case of GN associated with a cardiac pacemaker lead infection. The patient was treated with intravenous (IV) antibiotics, IV steroids, and the surgical removal of the cardiac pacemaker lead, resulting in the resolution of the glomerulonephritis and the return of the patient’s baseline kidney function in one week.

## Case presentation

Our patient, a 71-year-old female, came to the hospital due to several weeks of persistent weakness. Upon evaluation, she was found to have AKI with a serum creatinine of 9.3 mg/dL, leading to her admission. She has a past medical history of atrial fibrillation, chronic kidney disease stage 3b, coronary artery disease, hypertension, systolic heart failure with an ejection fraction of 25%, an automatic implantable cardioverter-defibrillator (AICD), and previously treated myelodysplastic syndrome now in remission. Her vital signs on admission included a blood pressure of 144/74 mmHg, heart rate of 66 beats/min, respiratory rate of 16/min, temperature of 36.4°C, and O_2_ saturation of 100%. Initial physical exam findings found the patient in no acute distress without jugular vein distention or lower extremity edema. Her lungs were clear to auscultation bilaterally, with an irregularly irregular heart rate without a murmur, and a soft, nontender abdomen. Admission laboratory values are seen in Table 1. Baseline hemoglobin was found to be 10.1 g/dL three months prior. Cryoglobulins were detected at 24 hours (positive), but not at 72 hours. The hepatitis panel, including the hepatitis C antibody titers, was non-reactive.

**Table 1:** Laboratory values on admission Erythrocyte Sedimentation Rate (ESR)

	Patient value	Reference
Complete Blood Count		
Hemoglobin (g/dL)	7.4	11.5-15.5
Hematocrit %	21.8	37-47
Platelets (10^3^/mcL)	78	150-400
Immunologic Studies		
C3 (mg/dL)	49.1	83-193
C4 (mg/dL)	16.2	15-57
ESR (mm/hr)	128	0-20

An electrocardiogram showed atrial fibrillation with new onset left bundle branch block and ST segment depressions in leads V_1_-V_3_ when compared to an earlier study. Blood cultures were obtained due to leukocytosis and they showed no growth after five days. Urinalysis demonstrated hematuria (+3) and proteinuria (+1). A urine culture was done due to the patient's complaint of dysuria, and it grew E. coli and Klebsiella. Consequently, the patient was started on IV ceftriaxone. Over the course of several days, the patient’s renal function gradually improved with a creatinine of 5.4 mg/dL after IV hydration therapy.

A transthoracic echocardiogram demonstrated an echogenic density adjacent to the right atrial segment of the pacing wire, measuring 1.9 cm x 1.0 cm, suggestive of a possible vegetation and moderate tricuspid regurgitation. A transesophageal echocardiogram showed vegetation on the AICD lead across the tricuspid valve. It was unclear whether or not the tricuspid valve itself was involved.

A kidney biopsy was performed to evaluate the etiology of the patient’s AKI and nephritic syndrome. The biopsy demonstrated a focal crescentic GN of an immune complex type, supporting a diagnosis of infection-related GN, most likely from a cardiac pacemaker lead infection similar to subacute bacterial endocarditis, and supported the need for lead removal. The vegetation was found to be isolated from the AICD lead. Aerobic and anerobic cultures of the vegetation did not grow an organism on the media.

Following the AICD lead removal, the patient was treated with IV vancomycin and continued on IV ceftriaxone for 6 weeks. This extended antibiotic regimen was prescribed to ensure thorough treatment and reduce the risk of mortality from a potential hidden infection, given her immunosuppressed state. The patient was started on IV steroids post-biopsy for two weeks, then transitioned to high-dose oral prednisone 1 mg/kg/day for eight weeks. The patient’s renal function gradually improved to its baseline creatinine of 2.2 mg/dL and did not require renal replacement therapy.

Pathological findings

**Figure 1:**
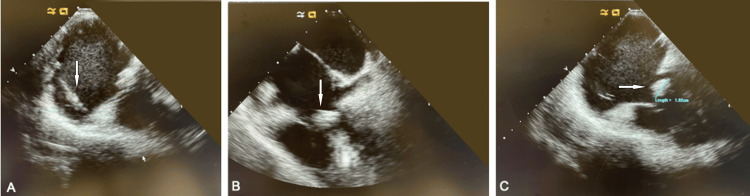
Transesophageal echography. (A) The pacing wire is visible in the right atrium. (B) The pacing wire is visible in the right ventricle. The vegetation is located across the tricuspid valve. (C) The vegetation measures 1.9 cm in length.

Transesophageal echography shows vegetation on the AICD lead at the level of the tricuspid valve and it is unclear whether there is any vegetation attached to the leaflet (Figures 1A-1C). There is moderate tricuspid insufficiency by color flow Doppler.

**Figure 2:**
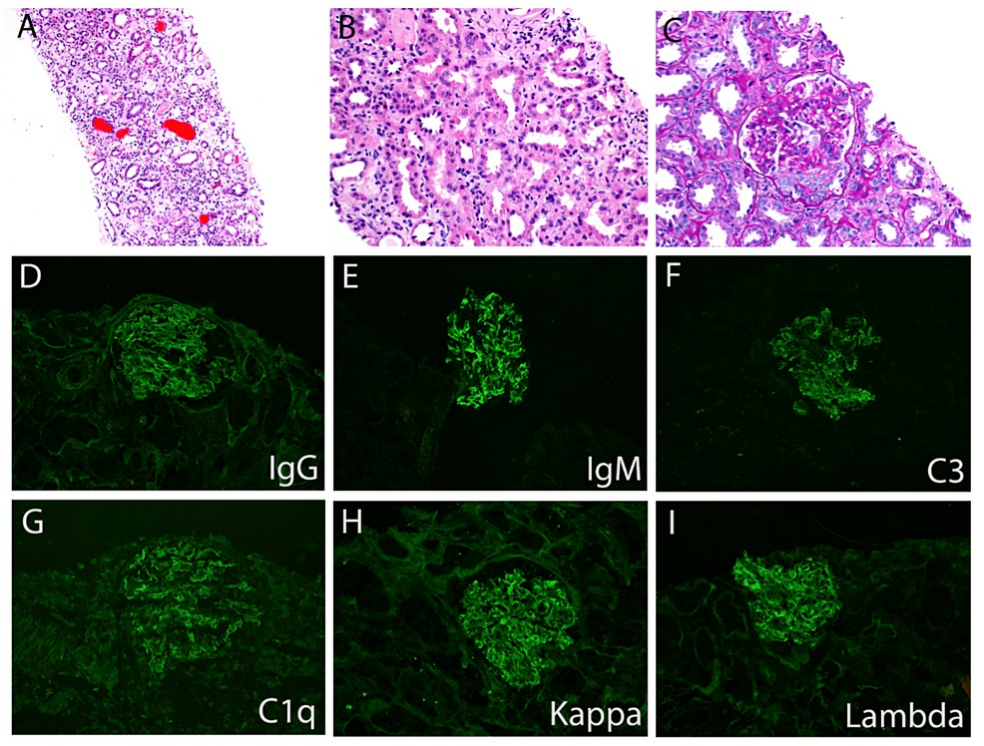
Kidney biopsy histopathology. (A) Hematoxylin and eosin (H & E)-stained section demonstrating renal medullar with interstitial edema and red blood cell casts, 200x. (B) H & E-stained section showing tubular injury and interstitial edema in renal cortex, 400x. (C) Periodic acid-Schiff stained section showing a glomerulus with mesangial matrix expansion and cellular crescent formation, 400x. (D-I) Immunofluorescence microscopy demonstrating granular mesangial staining for IgG (D), IgM (E), C3 (F), C1q (G), Kappa (H), and Lambda (I). All images are 400x magnification.

The renal biopsy adequately sampled both cortex and medulla and contained 27 glomeruli, of which 14 were globally sclerotic. Moderate interstitial fibrosis and tubular atrophy were present. The glomeruli demonstrated mesangial expansion with focal crescent formation, including both cellular and fibrous crescents (from prior active lesions). Immunofluorescence demonstrated granular mesangial staining for IgM (3+), C3 (2+), kappa (2+), and lambda light chains (2+), in addition to segmental fibrinogen staining in glomeruli with necrosis/crescent formation (Figures 2A-2I). The degree of IgM and C3 deposition was greater than typically seen in “pauci-immune” disease for ANCA-associated GN. Taken together, the biopsy demonstrated a focal crescentic GN with IgM-dominant immune complex deposition, consistent with endocarditis-associated GN.

## Discussion

The management of GN associated with IE depends on disease severity and response to antibiotic therapy [4-6]. While valve replacement surgery may be indicated in IE, there is no consensus on the management of GN associated with lead infections in regard to early surgical intervention.

Several studies suggest that surgical intervention with the removal of the vegetation eliminates the trigger for GN in IE [4-6,11,12]. However, further studies are warranted to examine the outcomes of surgical lead removal in lead-induced GN.

Our decision to remove the lead for the treatment of pacemaker-lead infection-associated GN was met with hesitancy. Current literature describes treatment protocols and their efficacy in the setting of native valve endocarditis-associated GN, not pacemaker-lead infection-associated GN [11-13]. Our patient had their pacemaker lead removed with the addition of antibiotics and corticosteroids to treat the GN. This patient was considered to have a low-virulence infection with an insidious onset, non-specific symptoms, and negative blood cultures. With the patient’s improvement after lead removal and without native valve repair or replacement, it was presumed that the infection was isolated to the pacemaker lead, and removal of the infected lead was necessary to eliminate the source of infection.

Based on our patient’s case, we recommend early surgical lead removal for cardiac pacemaker lead infection-associated GN. Limitations regarding our recommendation include a small sample size without control, resulting in the uncertainty that the GN would have been resolved with a prolonged course of IV antibiotics alone to treat the lead infection.

The presence of lead infection-associated GN in justifying the early removal of an infected cardiac pacemaker lead has not been established. This case may provide an opportunity for the nephrology, infectious diseases, and cardiology communities to explore the advantages of early lead removal and the course of GN in the presence of lead infection-associated GN.

## Conclusions

Cardiac pacemaker-lead infections are becoming more common given the increased use of devices, which mimic IE. Cases of infection-associated GN due to lead infections require prompt management. In our patient, early removal of the infected lead in conjunction with IV antibiotics and steroids led to clinical remission. As the use of cardiac devices continues to rise, we anticipate infection-associated glomerulonephritis to occur more frequently. We, therefore, urge others to report their management and outcomes of patients with this complication to gather data to develop guidelines for the treatment of lead infection-associated GN.
